# Fast Segmentation of Vertebrae CT Image Based on the SNIC Algorithm

**DOI:** 10.3390/tomography8010006

**Published:** 2022-01-03

**Authors:** Bing Li, Shaoyong Wu, Siqin Zhang, Xia Liu, Guangqing Li

**Affiliations:** 1 School of Automation, Harbin University of Science and Technology, Harbin 150080, China; 2020500046@stu.hrbust.edu.cn (S.W.); a501320776@163.com (S.Z.); liuxia@hrbust.edu.cn (X.L.); 2020510117@stu.hrbust.edu.cn (G.L.); 2 Heilongjiang Provincial Key Laboratory of Complex Intelligent System and Integration, School of Automation, Harbin University of Science and Technology, Harbin 150080, China

**Keywords:** simple non-iterative clustering, superpixel, medical image segmentation

## Abstract

Automatic image segmentation plays an important role in the fields of medical image processing so that these fields constantly put forward higher requirements for the accuracy and speed of segmentation. In order to improve the speed and performance of the segmentation algorithm of medical images, we propose a medical image segmentation algorithm based on simple non-iterative clustering (SNIC). Firstly, obtain the feature map of the image by extracting the texture information of it with feature extraction algorithm; Secondly, reduce the image to a quarter of the original image size by downscaling; Then, the SNIC super-pixel algorithm with texture information and adaptive parameters which used to segment the downscaling image to obtain the superpixel mark map; Finally, restore the superpixel labeled image to the original size through the idea of the nearest neighbor algorithm. Experimental results show that the algorithm uses an improved superpixel segmentation method on downscaling images, which can increase the segmentation speed when segmenting medical images, while ensuring excellent segmentation accuracy.

## 1. Introduction

Automatic image segmentation plays an important role in various image processing fields, which is a hot issue in the field of image processing. Image segmentation technology is the foundation of medical image processing, whether it is disease detection and classification, or three-dimensional reconstruction of organs. In the computer-aided diagnosis system, the automatic image segmentation will replace the doctor’s manual segmentation, saving manpower and material resources, and playing an important role in the diagnosis and treatment of diseases.

At present, there have been great developments in the field of medical image segmentation. Scholars have proposed many theories and methods for medical image segmentation, including based on traditional algorithms such as thresholds, region growth, level sets, and active contours. There are many methods [1,2,3,4,5] of segmentation recently, which have achieved good results in the segmentation accuracy, but most of them still have potential for improvement in segmentation speed. At present, the superpixel segmentation algorithm has developed in the image field [6] with a smaller calculation amount, faster-running speed, stronger anti-noise, and more robust, which is widely used in image segmentation and classification in various fields [7,8].

Superpixel algorithm was proposed in 2003 [9], which is a method of dividing pixels into blocks with consistent internal information. Superpixel segmentation algorithm is widely used in medical image segmentation due to its simple calculation and fast running speed. Then, based on the image, academics proposed the Normalized Cut Algorithm, the Meanshift Algorithm, the Watershed Algorithm, and other superpixel algorithms [10,11]. In 2012, Achanta et al. [12] proposed a simple linear iterative clustering (SLIC) algorithm based on the idea of clustering, which has the advantages of fast speed and simple calculation. On this basis, many superpixel algorithms have been proposed [13,14,15]. Achanta et al. [16] proposed a simple linear non-iterative clustering (SNIC) algorithm in 2017, which has better performance than the SLIC algorithm. In recent years, there have been many improvements and optimizations to the SNIC algorithm. Many methods [17,18,19,20] have been improved based on the original SNIC algorithm, and have been verified on various data and above, and their performance and practicability have been improved. However, many algorithms still have room for improvement in segmentation speed and segmentation accuracy.

In this paper, we propose an improved SNIC superpixel algorithm that is applied to medical image segmentation. It uses scale transformation to reduce the amount of calculation in the algorithm operation to save time and cost. At the same time, for the compact parameter problem of the selection algorithm and the complex texture of the medical image, the SNIC algorithm incorporates adaptive parameters and texture information to improve the segmentation effect of medical images. Therefore, the purpose of reducing the time required for superpixels to segment medical images and improving the accuracy of medical image segmentation is achieved.

In the Related Works section, the paper introduces the development of the superpixel segmentation algorithm; in the Materials and Methods section, the paper introduces the SNIC algorithm, the method we proposed, and the experimental environment and data set; in the Discussion section, we did comparative tests to prove The feasibility of the proposed algorithm. Finally, in the Conclusions section, a summary of the paper and the development direction of future image segmentation is made.

## 2. Related Works

### 2.1. Traditional Segmentation Algorithm

Jiang Qiulin et al. [1] proposed a segmentation algorithm based on the CV model and DRLSE model, which has a good segmentation effect for thyroid ultrasound images with blurring boundaries and uneven gray levels. Rehman et al. [2] proposed a new framework that combines traditional region-based level set algorithms and deep learning, which can accurately predict the shape of segmented vertebrae, and conducted experiments on multiple data sets, which has better performance in handling fracture cases. Chondro et al. [3] proposed a lung segmentation algorithm based on statistical area growth and adaptive graph cutting. Before segmentation, this algorithm determines the initial region through intelligent binarization and morphological operations, which can obtain higher segmentation accuracy with lower algorithm complexity. In addition to the above theories, methods such as bionics optimization and statistics are also widely used in medical image segmentation. Guerrout et al. [4] proposed an MRI image segmentation method combining hidden Markov random field and cuckoo algorithm. It is obtained better segmentation results with similar algorithms. Mostafa et al. [5] proposed a liver segmentation method for MRI images based on the Whale Optimization Algorithm (WOA). This method uses WOA algorithm to cluster and segment images by setting the number of categories. By testing this method on 70 sets of MRI images, the overall SSIM (Structural Similarity, which uses luminance, contrast, and structure to evaluate the similarity of two images) and SI (similarity index, which calculates the intersection ratio of pixel values to evaluate the similarity of two images) performance indicators of the experimental results reached 96.75% and 97.5%, respectively.

### 2.2. Superpixel Segmentation Algorithm

This section introduces the development history and research status of traditional segmentation algorithms and Superpixel segmentation algorithms.

Qin et al. [21] used a boundary-sensitive convolutional neural network based on superpixel to segment liver CT images. Accurate results could be obtained by marking superpixels as liver interior, liver boundary and nonliver background, constructing and training the CNN to predict liver boundary, and without complex post-processing. Tao Yongpeng et al. [22] improved the sensitivity of the activating contour model to the initial boundary, which used the Superpixel Algorithm to grid the image and then used the CNN to obtain the boundary superpixels as the initial contour. The approach of superpixel combined with machine learning is equally applicable to the segmentation and classification of medical images. Wu et al. [23] used the adaptive the SLIC Superpixel Algorithm and combined it with a support vector machine to train superpixel features, which can accurately segment gliomas and nerves. Glioma. Bechar et al. [24] proposed a semi-supervising superpixel by pixel glaucoma classification method, which established a glaucoma inspection scheme by labeling superpixels and training the classifier. Huang et al. [25] use the SLIC method to generate superpixels and classifiers to classify breast tumors, which can complete the segmentation and classification of breast tumors. After considering all indicators, they obtained better results than hand-drawn tumor contours. The above methods have been verified in the medical image data set, reflecting the applicability of the superpixel method in medical image segmentation and classification, which improves the running speed of the algorithm effectively.

Li et al. [17,18] proposed two improvement strategies based on the original SNIC algorithm framework. By introducing inter-pixel correlation, the accuracy of superpixel evolution was improved. At the same time, an optimized recursive method is proposed, which improves the computational efficiency of the algorithm, and has better segmentation performance than the original SNIC algorithm. Achanta et al. [18,19] proposed an optimized superpixel algorithm, which can generate superpixels of appropriate size in sequence, thereby automatically adapting to the local texture and scale of the image, and can obtain the result without affecting the segmentation performance too much. The most suitable number of superpixels. Xie et al. [19] proposed a segmentation algorithm based on superpixels and image tags. The algorithm uses the spatial distance and the number of image pixels to adaptively determine the number of superpixels and generate superpixels. Then, through non-neighbor joining and the most similar superpixels, the superpixels reach the preset number of image semantic tags, and automatically segment each image The semantic area of the target greatly improves the accuracy and efficiency of subsequent image processing. Aiming at the problem that the SNIC superpixel algorithm does not take into account the information contained in the image well, Bandara et al. [20] proposed a superpixel segmentation algorithm based on image information entropy. The image is divided into the information-rich area and the information-sparse area, and then the mean shift algorithm is used to generate the initial point center on the information-rich area, and the SNIC algorithm is used for segmentation. This allows the algorithm to segment more finely and densely in areas with rich information, and reduce superpixel segmentation in areas with less information. It can reduce execution time and unnecessary complexity during segmentation.

## 3. Materials and Methods

This article uses simple non-iterative clustering (SNIC) as the baseline. We introduced the SNIC algorithm in Section 1. We have added three parts: scale transformation, adaptive parameters, and texture information integration, which are described in Section 2, Section 3 and Section 4 of this section. Section 5 details the steps and process of the proposed method. Among them, the scale transformation reduces the amount of calculation, and the speed of the program that executes the segmentation task becomes faster. The addition of adaptive parameters improves the segmentation effect. The integration of texture information enables more unused useful features to be extracted by the algorithm, which enhances the robustness of the algorithm.

### 3.1. The SNIC Superpixels Algorithm

The SNIC algorithm has better performance than the SLIC algorithm. It is based on SLIC, assigning pixel labels to the shortest distance priority queue, and adopts the same distance measurement mode as that, which solves the problems of multiple iterations of SLIC, repeated calculation of overlapping local areas and pixel connection as post-processing execution, which effectively improves the efficiency of clustering. The algorithm adopts a non-iterative mode, which has the advantages of simple calculation, low memory consumption, and fast speed. Compared with other superpixel algorithms, it has the strong points of no subsequent area connection operations, no multiple iterations, fewer pixel accesses and distance calculations, and lower memory requirements.

Like SLIC, SNIC initializes the superpixel center in the regular grid of the image plane. The algorithm uses the same distance measure as SLIC, which combines normalized spatial and color distances. The spatial position is *x*, the color value is *c*, and the distance formula from the *j*th candidate pixel to the *k*th superpixel centroid is as follows:

(1) $$d_{j,k}=\sqrt{{\left(\frac{d_{x}}{s}\right)}^{2}+{\left(\frac{d_{c}}{m}\right)}^{2}}$$

where $d_{x}$ and $d_{c}$ are the spatial and color distances between the candidate point and the cluster center, respectively.

The calculation formula is:

(2) $$d_{x}=∥x_{j}-x_{k}∥$$

(3) $$d_{c}=∥c_{j}-c_{k}∥$$

where $x_{j}$ and $x_{k}$ are the location information of candidate points and cluster centers, $c_{j}$ and $c_{k}$ are the color information of candidate points and cluster centers, *s* and *m* are the normalization factors for spatial and color distances. For an image of *N* pixels, *K* superpixels, the value of *s* is $\sqrt{\left(N/K\right)}$. The value of *m*, also called the compactness factor, is set by the user.

Compared with SLIC, which requires multiple iterations to converge superpixel centers, SNIC can update cluster centers online in one iteration. Starting from the initial seed point, the SNIC algorithm uses a priority queue to select the next pixel to add to a superpixel.

On the regular grid of the image, we get the initial *K* seeds *C*[*K*] = {$x_{k}$, $c_{k}$}. Using these seed pixels to create *k* elements $e_{i}$ = {$x_{i}$, $c_{i,k}$, $d_{i,k}$}, where each label *k* is set as a unique superpixel label. From 1 to *K*, each distance value $d_{i,k}$, which represents the distance from the pixel to the *K*th cluster center is set to 0. The priority queue *Q* is initialized by these *K* elements. When taken out, *Q* always returns element $e_{i}$ from the *K*th cluster center with the smallest distance $d_{i,k}$.

While *Q* is non-null, the smallest element of $d_{i,k}$ is popped. If the pixel position on the label graph *L* is marked by the element is not labeled, it is given the label *k* of the element. The centroid value is the average value of all the pixels in the superpixel, which is updated with this pixel. What’s more, we will create a new element for its 4 or 8 neighboring pixels, assign a label *k* and calculate $d_{i,k}$, and then fill these new elements into the priority queue *Q*.

When the algorithm is executed, one end of an empty priority team will be assigned a label, and the other end will be filled with new candidates. The algorithm will be terminated when there are no remaining unmarked pixels to add new elements to the queue, and the queue has been emptied.

### 3.2. The Scale Transform

This paper improves the SNIC algorithm, using the downscaling method and K-Nearest Neighbor (KNN), that is, each sample can be represented by its *K* nearest neighbor values.

**Figure 1:**
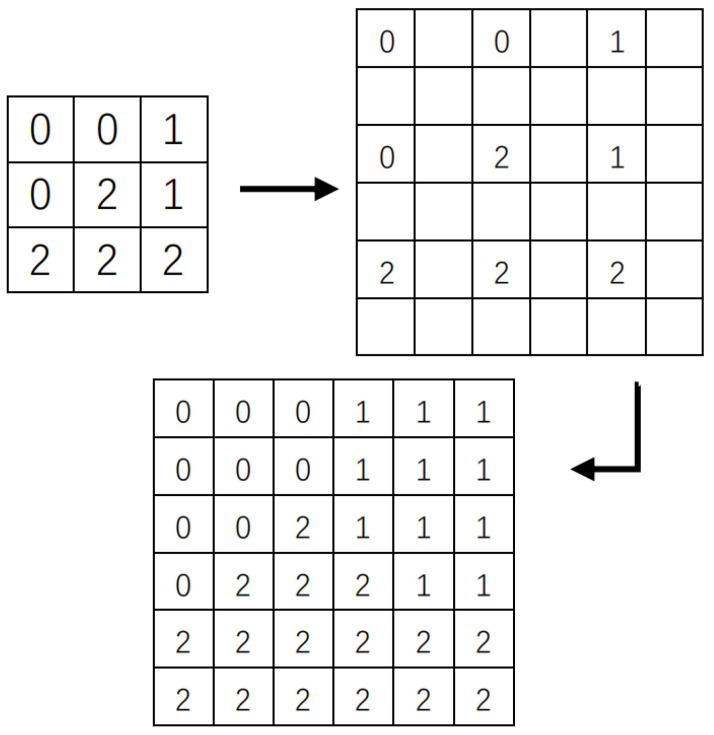
From downscaling to original scale classification.

This method can reduce the amount of calculation by reducing the number of pixels, so that improves the segmentation speed of the algorithm.The following steps will introduce the implementation process of the ruler change algorithm:

1. Superpixel segmentation is performed on the downscaling image with 1/4 pixels after removing the pixels of odd rows and odd columns;
2. Restored the labeled map to the original scale by using the KNN algorithm Figure 1, which based on the segmentation label map of the reduced scale image;
3. Complete the superpixel segmentation of the original image, by classifying the labels of the pixels in the original image according to the superpixel marking a map of the original scale.

**Figure 2:**
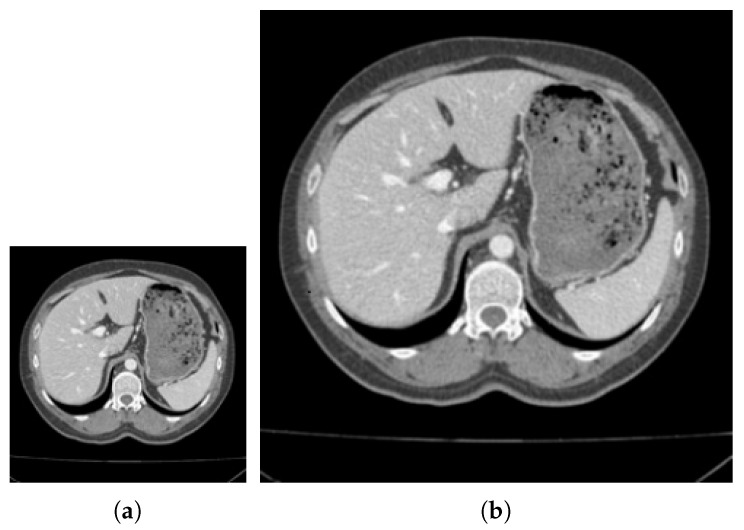

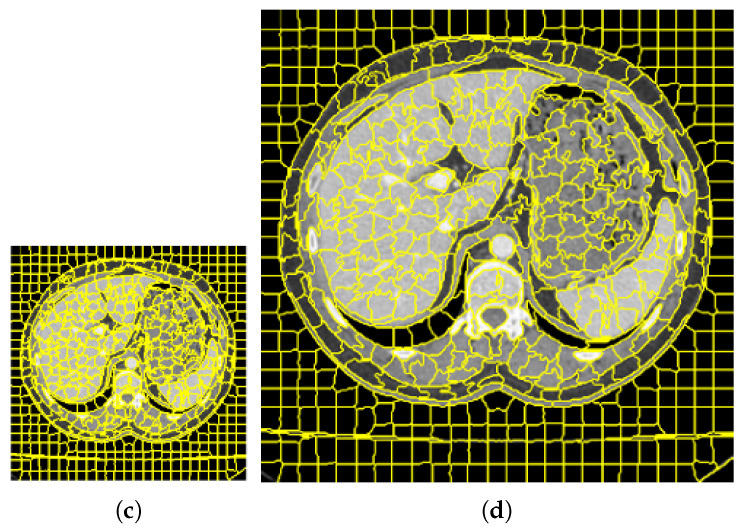
The results of downscaling segmentation and restoring to the original scale. (**a**,**c**) represent the pictures after downscaling, respectively, (**b**,**d**) represent the pictures restored to the original scale, (**a**,**b**) are the original images of the label image, (**c**,**d**) are the pictures obtained after clustering.

Figure 2 shows the segmentation results of the downscaling image using the improved SNIC algorithm and the label image restored to the original scale with the KNN idea. The internal information loss of the segmentation target caused by downscaling will not affect the segmentation results of the original scale. Because the principle of the superpixel algorithm is to cluster the pixels in the image into several superpixel blocks according to the similarity between pixels, the missing pixels within the superpixels segmented on the downscaled image will still be in the corresponding superpixels on the original scale image. Although the downscaling method causes certain edge information loss, the segmentation effect is not affected after the SNIC algorithm is used to segment and restore to the original scale, and it can be seen from the Figure 2 that the same downscaled image can still be obtained after restoring to the original scale image with consistent and complete segmentation results.

### 3.3. Adaptive Parameters

In this paper, we present an improved version of the SNIC algorithm that overcomes the following limitations of the maximum color distance value *m*: (1) the original algorithm needs to select parameters manually; (2) the parameters cannot well adapt to the differences of different local areas. We optimize the parameter *m* in the algorithm to make it an adaptive parameter, which can be dynamically changed with the local characteristics during the operation of the algorithm so that appropriate parameters can be used for calculation. It can use different parameters for calculation according to the difference of each superpixel, which can better distinguish different regions, improving the effect of segmentation.

The details of the improvement ideas are as follows: for each superpixel block, when a new pixel is added, the parameter *m* is the difference between the maximum gray distance 255 and the average gray distance between each pixel and the center of the superpixel. The parameter *m* calculation formula is:

(4) $$m=255-\frac{1}{n}\sum_{j=1}^{n}\sqrt{{\left(c_{j}-c_{k}\right)}^{2}}$$

As is known to us that the superpixel internal pixels tend to be consistent. The distribution of the values obtained by Equation (4) is basically in the range of 200 to 255 with a small gap. To make this parameter have a greater impact on the calculation process, this article uses the *Gamma* transformation method to transform it, and the transformed formula is:

(5) $$m=255\cdot {\left(\frac{255-\frac{1}{n}\sum_{j=1}^{n}\sqrt{{\left(c_{j}-c_{k}\right)}^{2}}}{255}\right)}^{\gamma }$$

where $c_{j}$ and $c_{k}$ are the pixel values of the joined pixels and the superpixel center, respectively, *n* is the number of pixels in the superpixels, and γ is the *Gamma* transformation parameter. After *Gamma* transformation, the distribution of parameter values is stretched to a larger interval range, the gap between parameters is larger, and the effect resulting from parameter changes is more pronounced.

Through the above improvements, the superpixels are more compact when calculating the grayscale uniform pixels, and more sensitive to the edge pixels the growth of the superpixels when calculating the grayscale unevenness and boundary area, making the superpixel boundary closer to the real edge.

**Figure 3:**
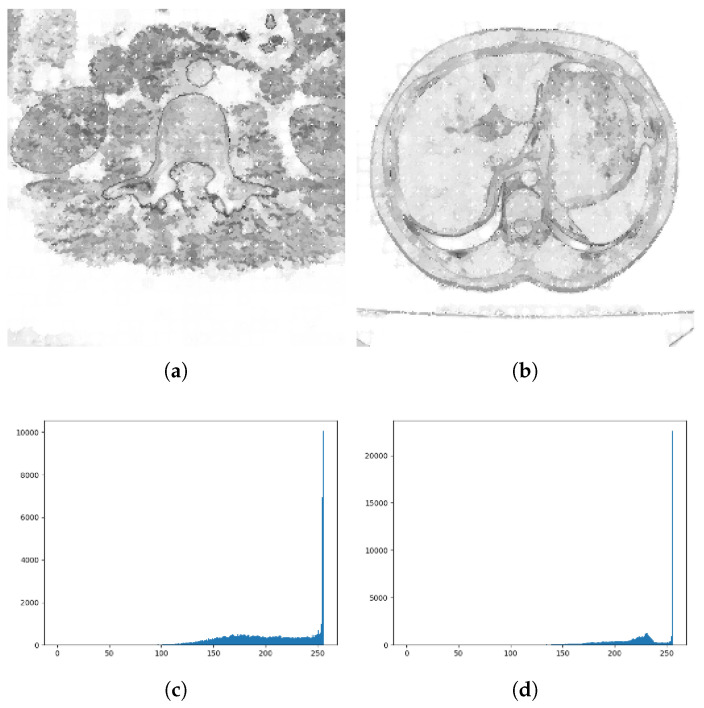
Grayscale image and corresponding parameter statistical histogram. (**a**,**b**) represent the grayscale image after adaptive parameter transformation. The white dot in the image represents the center of the super pixel; (**c**,**d**) represents the grayscale histogram of this image.

In order to show the effect of adaptive parameters more clearly, Figure 3a–d respectively show the adaptive parameter value map and its grayscale histogram used when calculating every pixel. Because pixels with similar gray values tend to be clustered into one class when superpixels are assembled. As can be seen from the adaptive parameter histogram shown in Figure 3c, the parameter values are mainly concentrated in the high-value interval. There are more black background superpixels in the image, so that the internal gray level similarity is high, with a lower gray difference gray level. According to Equation (5), most of the adaptive parameter values are close to the maximum value of 255, so when adding new pixels, the weight of gray similarity calculation is smaller, and the weight of spatial position is larger. Therefore, superpixels are more regular and compact in the gray-scale flat area. Moreover, it can be seen from Figure 3c,d that the adaptive parameter changed greatly value at the edge position. It shows that when the SNIC superpixel grows to the edge pixel, the gray distance weight would increase, and the edge will have a greater impact on the segmentation of the superpixel, which helps to improve the image segmentation effect.

### 3.4. Integrating Texture Information

The texture is very important for medical images, which can reflect the characteristics of tissue structure. Therefore, this paper uses the Local Binary Pattern (LBP) algorithm to extract the texture information of the image and add it to the calculation of the SNIC algorithm to solve the problem that the latter considers the spatial location information and color information but lacks the texture information of the image. The LBP feature extraction algorithm has a fast calculation speed, with advantages of rotation invariance and grayscale invariance. It is suitable for medical images such as images with large local texture differences and irregular grayscale distribution.

**Figure 4:**
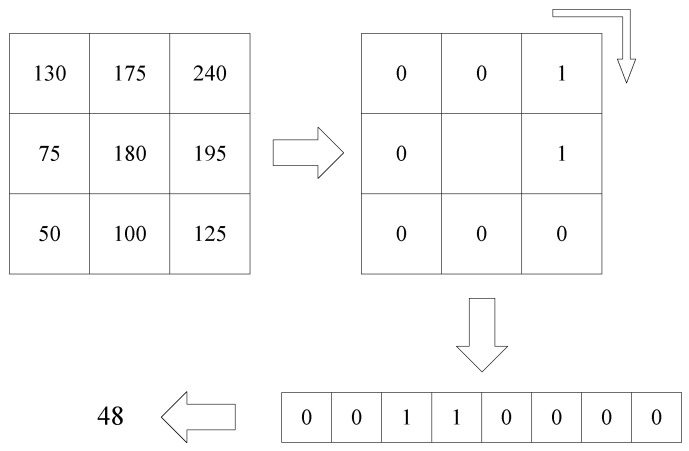
The principle of LBP algorithm.

The LBP algorithm builds feature maps from inter-pixel gray value comparisons. Comparing the size of the pixel 8 adjacent gray values with the center gray value, the neighborhood pixels with values greater than the center were labeled 1, those with values less than the center were labeled 0. Through this thresholding method, the neighborhood pixel is processed into a binary value, and then it can be converted into a decimal value through the conversion from binary to decimal. This value is the LBP characteristic value of the point. The principle of the LBP algorithm is shown in Figure 4:

The calculation formula of LBP characteristic value is:

(6) $$LBP=\left(x_{c},y_{c}\right)=\sum_{P=0}^{P-1}2^{P}S\left(i_{P}-i_{c}\right)$$

where ($x_{c}$,$y_{c}$) is a central pixel, $i_{c}$ and $i_{p}$ are the gray values of the central pixel the neighborhood pixels, respectively, and *s* is the following function:

(7) $$s\left(x\right)=\left\{\begin{array}{cc} 1 & x⩾0\\ 0 & x<0 \end{array}\right.$$

The distance formula of the improved SNIC algorithm by adding texture features as following:

(8) $$d_{j,k}=\sqrt{{\left(\frac{d_{x}}{s}\right)}^{2}+{\left(\frac{d_{c}}{m}\right)}^{2}+{\left(\frac{d_{t}}{t}\right)}^{2}}$$

The $d_{t}$ formula as:

(9) $$d_{t}=\sqrt{{\left(t_{j}-t_{k}\right)}^{2}}$$

where $t_{j}$ and $t_{k}$ respectively represent the texture value of the *j*th candidate pixel and the *k*th cluster center extracted by the LBP algorithm, and *t* represents the normalization factor of the texture distance.

### 3.5. Improved SNIC Algorithm Steps

1. To obtain the feature map by extracting the image texture features, then reduce its scale;
2. Set the parameter *t*, the number of superpixels *K*, and distribute the seed point position on the downscaled image;
3. Create a blank label image *L* with the same size as the downscaled image, and initialize the priority queue *Q* with the element created by the seed point $e_{i}$ = {*$x_{i}$,*$c_{i}$$t_{i}$,k,$d_{i,k}$ = {0,0,0,0,0};
4. Take out the smallest element of $d_{i,k}$ from *Q*. If it is not marked at the same position in the marked image *L*, which will be marked as *K*;
5. Calculate the average value of all pixels in the superpixel to update the center of the superpixel. Then, calculate and update the adaptive parameter *m* according to Equation (5);
6. Calculate $d_{i,k}$ for the unmarked pixels in the 4 or 8 neighbors according to Equations (8) and (9). Create a new element and assign the label *k*, and fill it in *Q*;
7. If *Q* is non-empty, switch to Step4, otherwise switch to Step8;
8. Obtain the segmentation results by restoring the labeled map *L* to the original scale, with the KNN algorithm shown in Figure 1.

**Figure 5:**
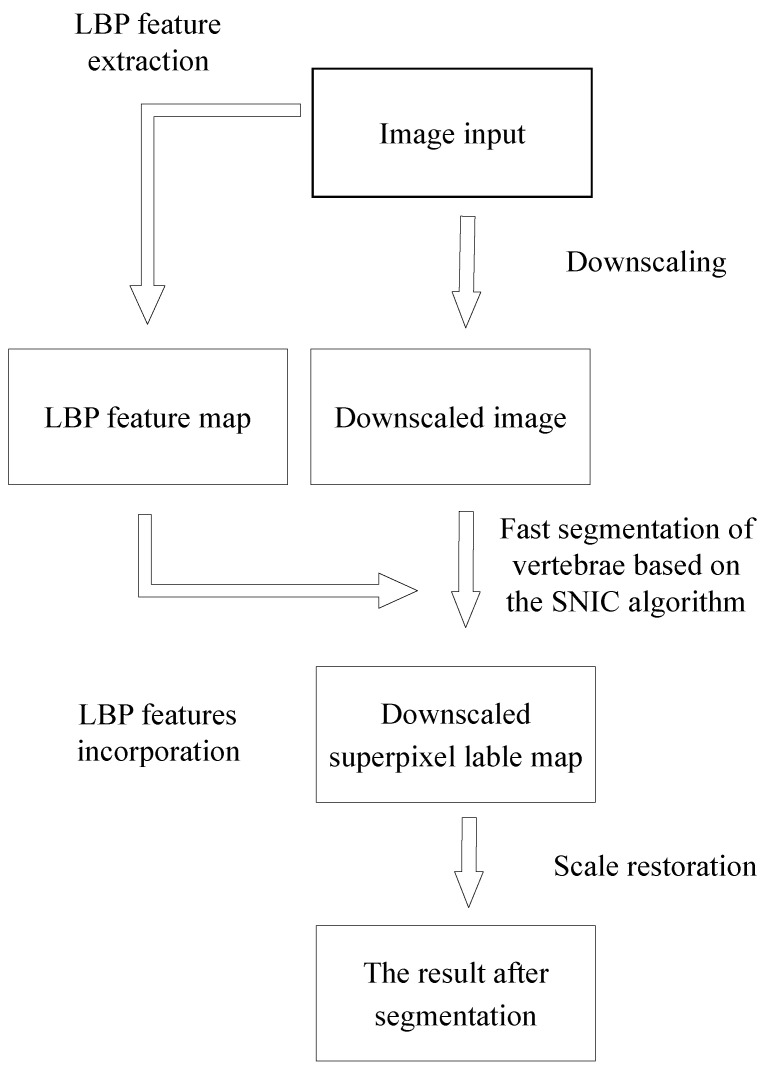
Improved SNIC algorithm flow chart.

The process of the improved SNIC algorithm is shown in Figure 5. Firstly, the process of extracting feature maps and downscaling from the input image is used to obtain a downscaled image; Secondly, the downscaling superpixel marker map is obtained by using the improved SNIC algorithm combining adaptive parameters and LBP feature values in downscaling; Finally, with the help of the KNN algorithm, labeling the superpixel on the input image, we can get the segmentation result by restoring the marked image to the same scale as the input.

## 4. Experiments

**Figure 6:**
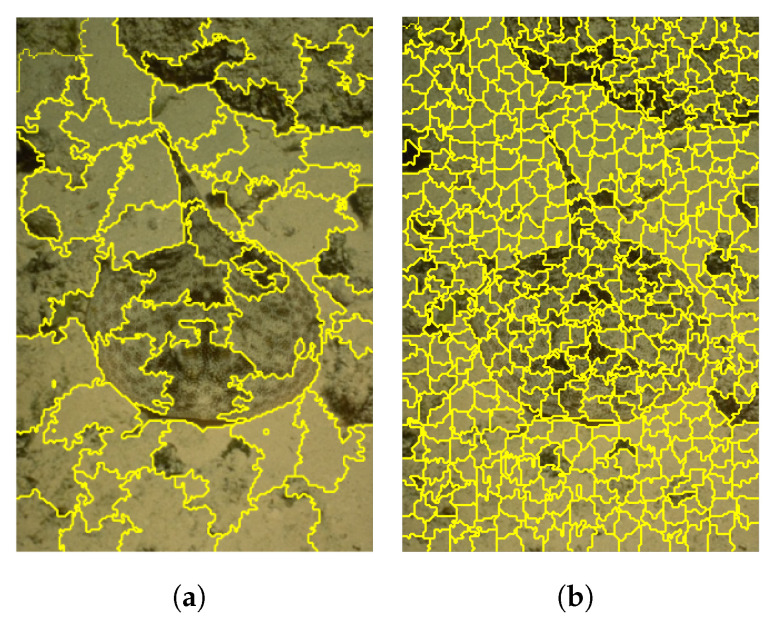
Comparison of segmentation effect with different superpixel numbers. (**a**) is the divided image when the number of super pixels k = 50, and (**b**) is the divided image when the number of super pixels k = 500. (**a**) k = 50. (**b**) k = 500.

**Figure 7:**
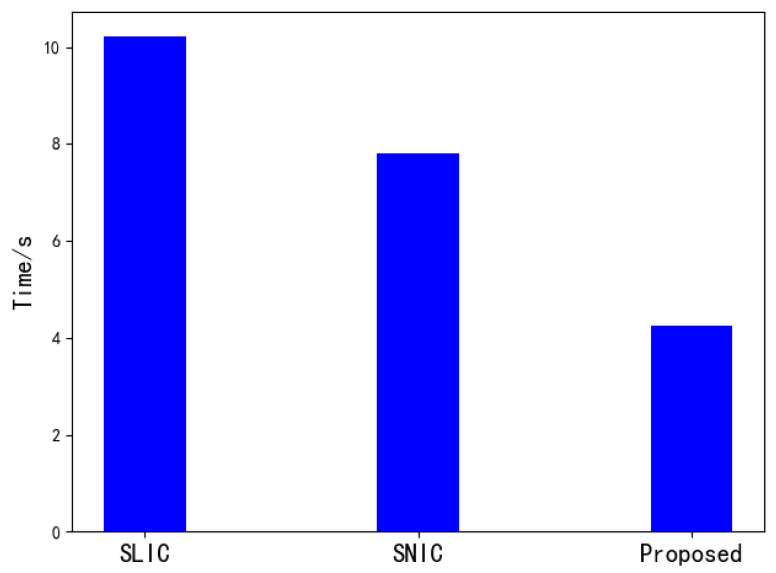
Comparision of segmentation time.

**Figure 8:**
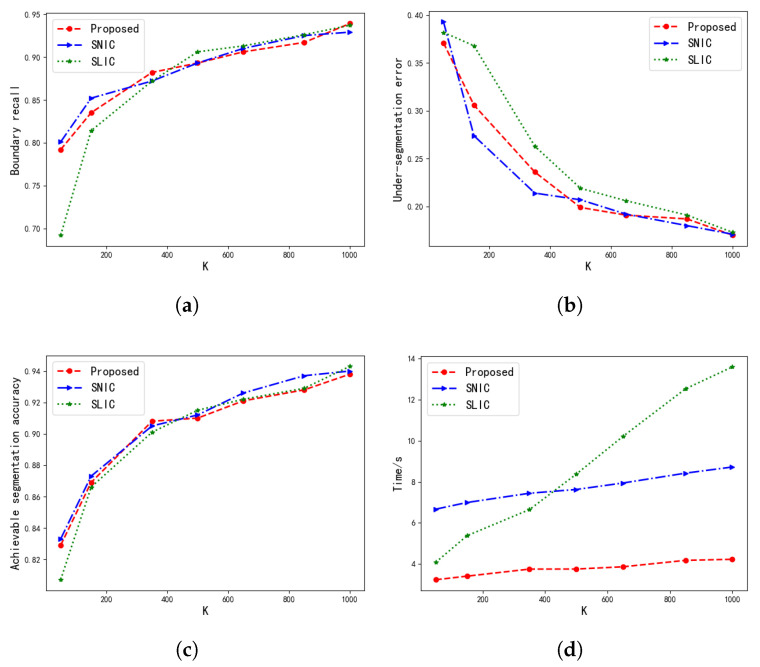
Performance analyzing. As the number of superpixels increases, the performance of the three methods is compared. (**a**) is the boundary recall (*BR*) line chart, (**b**) is the under-segmentation error (*USE*) line chart, (**c**) is the achievable segmentation accuracy *(ASA*) line chart, and (**d**) is the line chart representing the time required by the algorithms.

**Figure 9:**
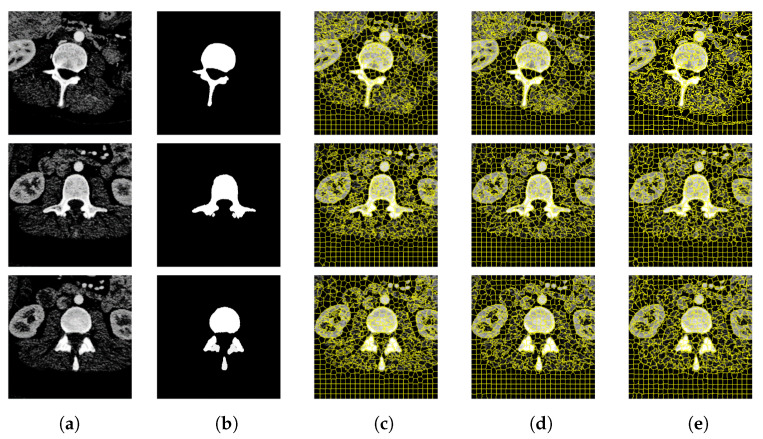
CT image segmentation of vertebrae. (**a**) Original image. (**b**) True image. (**c**) SLIC. (**d**) SNIC. (**e**) Proposed.

**Figure 10:**
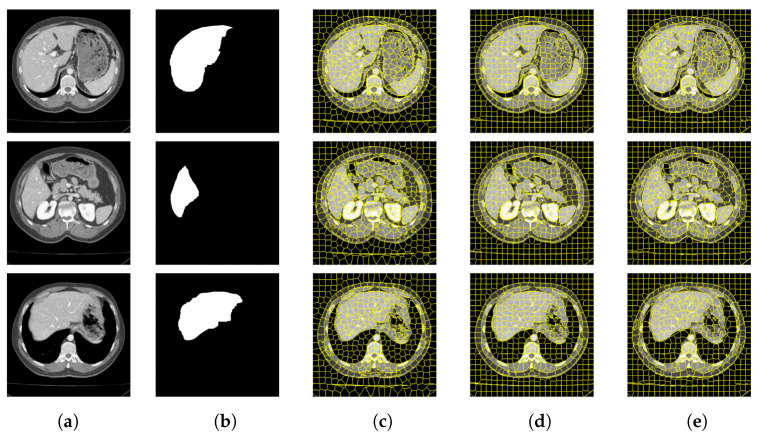
CT image segmentation of liver. (**a**) Original image. (**b**) True image. (**c**) SLIC. (**d**) SNIC. (**e**) Proposed.

**Figure 11:**
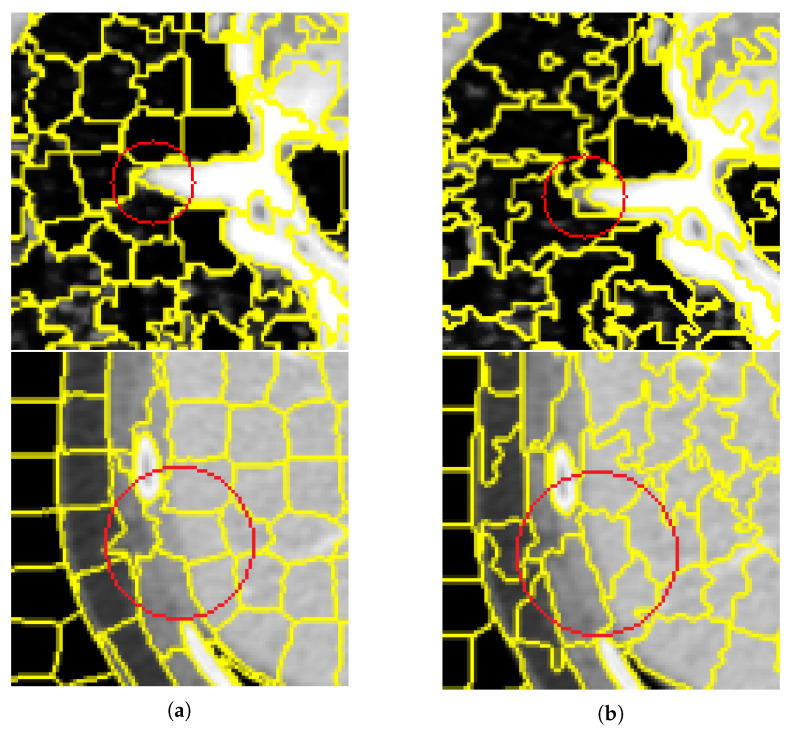
Comparison of segmentation results between SNIC algorithm and the algorithm proposed in this article. (**a**,**b**) are the super-pixel segmentation results and the corresponding detailed images on the vertebral CT images using the SNIC and our proposed method, respectively. (**a**) The SNIC algorithm. (**b**) Proposed algorithm.

In this section, this article conducts experiments on the Berkeley Segmentation Data Set and the medical CT image data set. Experiment on the former data set to explore the influence of the number of different superpixels on the experimental results, so as to determine the number of superpixels set in the experiment on the latter data set. In Figure 6 and Figure 7 and Table 1 and Table 2 respectively show that compared with other algorithms, the proposed method has a significant reduction in the image segmentation time and ensures the segmentation performance. Figure 8 shows the performance comparisons between the algorithm proposed in this paper and other algorithms as the number of superpixels grows. In addition, Figure 9, Figure 10 and Figure 11 show that the proposed method can extract more texture details, so that more closely fits the real edge details of the vertebrae. At the end of this section, the paper introduces the experimental environment and data set.

### 4.1. Exploration of the Feasibility and Effectiveness of the Proposed Method

To prove the feasibility and effectiveness of the algorithm in this paper, we have successively experimented on the Berkeley Segmentation Data Set and calculated the segmentation time and evaluation coefficient to that compare the segmentation results of the algorithm and the performance of the algorithm. We evaluate the performance of the algorithm by calculating boundary recall (*BR*) [26], under-segmentation error (*USE*), and achievable segmentation accuracy coefficients (*ASA*). The above three evaluation methods are all calculated on the edge of the image. *BR* is a measure of the score that the true boundary is correctly restored by the superpixel boundary. *USE* measures the pixels leaked from the true boundary. *ASA* is used as the highest achievable object segmentation accuracy when using superpixels as the unit [27]. The definition of the three evaluation functions are shown in the following formulas:

(10) $$BR=\frac{TP}{TP+FN}$$

(11) $$USE=\frac{FP}{TP+FP}$$

(12) $$ASA=\frac{TP}{\sum G_{i}}$$

where *BR* is the proportion of ground truth edges that fall within a certain distance *d* of at least one superpixel boundary. We use *d* = 2. Given a real boundary image *G* and algorithm boundary image *B*. True (*TP*) represents the boundary pixels in *B* in the boundary range *d* existing in *G*, and false negative (*FN*) represents the pixels in the boundary image *B* that do not exist in the boundary range *d* of *G*, which actually belong to the boundary range of the boundary image *G*. False positive (*FP*) means that the boundary pixels in the boundary image *B* that do not exist in the boundary range d of *G* actually belong to the boundary range of *G*. $\sum G_{i}$ represents the sum of all pixels within the boundary of *G*.

During the experiment, we found that the setting of the number of superpixels *K* has a certain impact on the segmentation effect and segmentation time. As shown in Figure 6 below, the difference in the number of superpixels produces different results. When the number of superpixels is set to 50, the segmentation of some edge parts is not accurate with a phenomenon of under-segmentation. While the number of superpixels is set to 500, the edge blurred area can be well segmented, which can improve the segmentation effect of the image to a certain extent. However, too many superpixels may lead to over-segmentation. Therefore, it needs to determine the specific number of super mixing according to the needs of practical applications.

Figure 7 is a comparison of the running time of the three algorithms when the number of superpixels is 1000 on a 512 × 512 image. It can be seen that when the number of superpixels is larger, the SNIC algorithm is slightly faster than the SLIC algorithm in segmentation speed. This paper uses a way of downscaling to improve the speed by reducing the amount of calculation based on SNIC, which is better than the SNIC and SLIC algorithm in segmentation speed.

Figure 8 shows the performance comparison of our algorithm, SLIC, and SNIC. In the figure, the *x*-axis represents the number of superpixels *K*, the *y*-axis represents BR, USE, and ASA coefficients and the segmentation time, and the three lines represent the three methods respectively. Compared with SLIC and SNIC, the performance index of this algorithm is showing no difference on the Berkeley Segmentation Data Set. From the results of the segmentation time, it can be seen that the segmentation speed of SLIC is slightly faster than that of SNIC when the number of superpixels is small. But, as the number of superpixels increases, the segmentation time of SLIC is gradually greater than that of SNIC. At the same time, it can be seen that the improved SNIC algorithm in this paper greatly reduces the computational complexity of the algorithm. Compared with the SLIC and SNIC algorithms, the segmentation time has been greatly improved.

### 4.2. Verification of Medical CT Images with Proposed Method

Figure 9 and Figure 10 are the segmentation results on the CT image of the vertebrae and the CT image of the liver, respectively. Although this article uses downscaling in the process of improving the algorithm to reduce the number of pixels, which caused a certain degree of edge information loss. It can be seen that the segmentation effect of the improved SNIC algorithm is similar to the original algorithm. Compared with SLIC and SNIC, the algorithm in this paper is more sensitive to texture features and can segment edges accurately, which is more practical.

Figure 11 is a detailed view of the segmentation results of the original SNIC algorithm and the improved SNIC algorithm in this article. From the detailed view, it can be seen that for the fuzzy edge parts, the improved SNIC algorithm added texture features and adaptive parameters, which were closer to the real edge of the vertebrae. It can be shown that the algorithm in this paper not only overcomes the impact of the lack of information caused by using downscaling segmentation results when segmenting CT images of vertebrae but also can obtain better results for fuzzy edge regions.

In terms of segmentation accuracy on medical images, this experiment uses three superpixel algorithms to segment the experimental images. The final results were compared with the doctor’s manual segmentation results, and *Dice*, *Jaccard*, and the *CCR* coefficients were calculated to evaluate the segmentation results.

As for the accuracy of the system, this article used three evaluation indicators: *Dice*, *Jaccard*, and Correct Classification Ration (*CCR*) for experimental evaluation. The Dice coefficient is mainly used to calculate the similarity between the predicted result and ground truth. *Jaccard* is concerned with the issue of whether the common characteristics of individuals are consistent, and is used to compare the similarities and differences between sample sets. *CCR* is used to indicate the proportion of the number of pixels correctly segmented by the algorithm to the number of pixels in the ground truth. The three quantitative evaluation indicators are defined as follows:

(13) $$Dice=2\frac{\left|SEG\cap GT\right|}{\left|SEG+GT\right|}$$

(14) $$Jaccard=\frac{\left|SEG\cap GT\right|}{\left|SEG\cup GT\right|}$$

(15) $$CCR=\frac{n}{N}$$

where, *SEG* and *GT* respectively are the result of automatic algorithm segmentation and the standard result of expert segmentation, *n* is the number of correctly segmented pixels, and *N* is the total number of image pixels. The values of the three index coefficients range from 0 to 1. The closer to 1, the better the segmentation effect.

**Figure 12:**
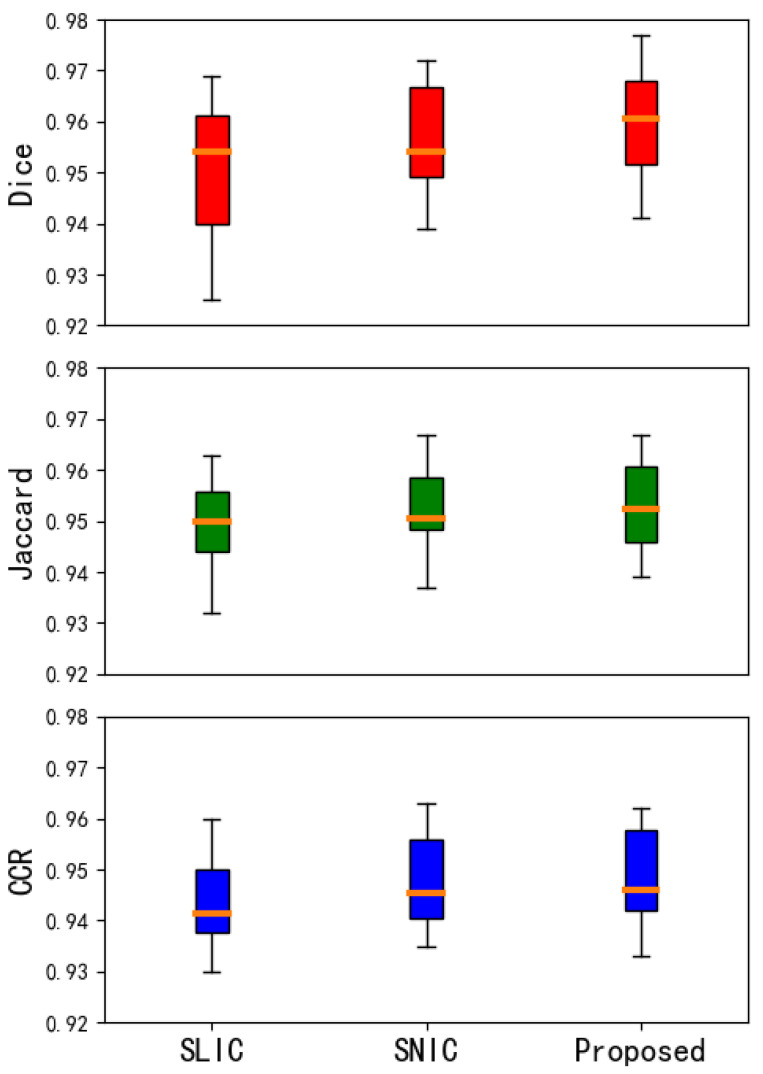
Box chart of evaluation index. The above three pictures respectively *Dice*, *Jaccard*, and Correct classification ration (*CCR*) of SLIC (first column), SNIC (second column) and Proposed method (third column), respectively.

In this article, the segmentation accuracy of different algorithms is evaluated by calculating the evaluation coefficients of different segmentation algorithms on the vertebral body image. We can understand the performance of the algorithm more intuitively. This article uses multiple pictures for experimental verification. After data statistics, the result evaluation coefficient obtained is shown in Figure 12 below:

The middle line of the box plot represents the median, indicating the average level of the evaluation coefficient; the upper and lower limits of the box are the upper quartile and the lower quartile of the evaluation coefficient, reflecting the fluctuation of the data to a certain extent Degree; there is a line above and below the box, which represents the maximum and minimum values of the evaluation coefficient. In this paper, the improvement based on the SNIC algorithm is better than the SLIC algorithm and SNIC algorithm in the overall distribution of the performance of segmentation of vertebral CT image, which can adapt to the characteristics of medical CT and get satisfactory results on medical CT image.

Table 1 and Table 2 show the comparison of evaluation coefficients on vertebral CT data and liver CT data respectively. It can be seen that the improvement of the SNIC algorithm in this paper can ensure that the segmentation accuracy of medical CT in the segmentation of medical images does not decline, and the segmentation speed has been significantly improved. Compared with SLIC and SNIC, the segmentation time is saved by 60% to 70%.

### 4.3. The Experimental Environment and Data Set

This experiment is implemented on Intel (R) Core (TM) i5-4590 CPU, 3.3GHz, 8G memory, AMD radon (TM) graphics card, and Windows10 platform. The segmentation experiment was done on the *Berkeley Segmentation Data Set* and the medical CT image data set.

## 5. Discussion

This paper proposes a fast segmentation of vertebrae CT image based on the SNIC algorithm. The article proposes three aspects of improvement, namely The scale transform, adaptive parameter, and integrating texture information. With the help of scale transform, the amount of calculation is reduced, which speeds up the execution of the algorithm. The reduction in the amount of calculation will affect the performance of image segmentation. This article takes two measures, adaptive parameter and integrating texture information.

This article takes two measures, adaptive parameter and integrating texture information. The addition of adaptive parameter makes the parameter setting independent of manual setting. In the image of the vertebrae after adaptive parameter processing, the edges of the vertebrae occupy a larger weight, resulting in a better superpixel segmentation effect. Integrating texture information is a method that adds texture information when extracting features, not just using space and color information. After adding texture information, the proposed method can reflect the characteristics of a certain tissue area to a certain extent and improve the segmentation performance.

In Table 1 and Table 2, we can see that when segmenting vertebral images, compared with SNIC, the proposed method of *Dice*, *Jaccard*, and *CCR* are all higher by 0.3%, but the time is reduced by 6.2 s; in segmentation In the liver image, the Dice, Jaccard, and CCR of the proposed method are 0.2%, 0.7%, 0.8% higher, respectively, and the time is shortened by 8.3 s, which is one third of the time used by the SNIC method.

Although the proposed method has no significant increase in the evaluation coefficient of segmentation, it can be clearly found from Figure 11 that the proposed method is more suitable for the detailed segmentation of vertebrae, and the regions of the same category are clustered together, which has a good segmentation effect. And faster image segmentation speed.

## 6. Conclusions

This paper proposes an improved SNIC medical image segmentation algorithm. This paper adopts the following improvements: 1. Reduce the amount of calculation by downscaling; 2. Incorporate the texture information into the original SNIC algorithm, improve the segmentation accuracy of complex regions of texture; 3. Propose an adaptive parameter, save the process of manually selecting parameters enables the algorithm to dynamically set adaptive parameter values according to the characteristics of the local area, which improves the overall segmentation effect. Experimental data on image segmentation data sets and medical images show that the proposed algorithm consumes less time, with a higher segmentation accuracy, and is feasible and practical, compared with the SLIC algorithm and the original SNIC algorithm. In the following research, we will focus on testing experiments on more types of images and research in the direction of combining with deep learning methods. At the same time, it will continue to optimize and improve the algorithm’s accuracy and efficiency, so that this method can play a greater role in image processing in various fields.

## Figures and Tables

**Table 1:** Comparison of evaluation coefficients of vertebrae segmentation.

Method	Dice	Jaccard	CCR	Time
SLIC	0.951	0.933	0.935	12.9 s
SNIC	0.971	0.952	0.958	10.2 s
The algorithm in this paper	0.974	0.955	0.961	3.8 s

**Table 2:** Comparison of evaluation coefficients of liver segmentation.

Method	Dice	Jaccard	CCR	Time
SLIC	0.944	0.949	0.951	14.3 s
SNIC	0.965	0.958	0.962	12.5 s
The algorithm in this paper	0.967	0.965	0.970	4.2 s

## Data Availability

Data is contained within the article.

## Abbreviations

The following abbreviations are used in this manuscript:

SSIM	Structural Similarity Index Measure
SI	Similarity Index
KNN	k-NearestNeighbor
SLIC	Simple Linear Iterative Cluster
SNIC	Simple Non-Iterative Clustering
LBP	Local binary pattern
BR	Boundary Recall
USE	Under-Segmentation Error
ASA	Achievable Segmentation Accuracy
SEG	the result of automatic algorithm Segmentation
GT	Ground Truth
CT	Computed Tomography
CCR	Correct Classification Ration
